# An observational descriptive cross sectional multicenter study of health related quality of life among Iraqi patients after total hip replacement

**DOI:** 10.1016/j.amsu.2019.10.031

**Published:** 2019-11-12

**Authors:** Mahmoud Khudair Yaseen, Faiq I. Gorial

**Affiliations:** a Department of Surgery, College of Medicine, University of Baghdad, Baghdad, Iraq; b Department of Medicine, College of Medicine, University of Baghdad, Baghdad, Iraq

**Keywords:** Health related quality of life, Harris hip score, Total hip replacement

## Abstract

**Background:**

Hip replacement is highly effective procedure to decrease pain and disability in patients with hip arthritis and accordingly can affect health related quality of life (HRQOL). Globally, limited studies have reported impact of total hip replacement (THR) on HRQOL and there is no previous reports of HRQOL among Iraqi patients after THR.

**Objective:**

To evaluate HRQOL in patients after THR and to assess impact of sociodemographic characteristics on it if present.

**Patients and methods:**

A multicenter cross sectional study was conducted on 96 patients with THR in Iraq. Sociodemographic characteristics were measured. HRQOL after THR was evaluated using Harris hip score (HHS).

**Results:**

The mean age of patients was 56.76, (13.88) years with a range of 23–90 years. Most of patients were females (52 patients (54.2%). Mean BMI was 44.87(8.07) kg/m^2^ with a range of 28.1–56.7 kg/m^2^. The mean(SD) of HHS was 84.39 (7.25) with minimum score of 61.7 and maximum score 93.8. Sociodemographic characteristics had no statistically significant effect on HRQOL measured by HHS except BMI. For each 1 unit increase in BMI, there is significantly and independently decrease in HHS by −0.276.

**Conclusions:**

THR improved HRQOL. BMI was the only significant independent factor that was negatively correlated with HRQOL.

## Highlights

• Total hip replacement improved health related quality of life.
• BMI was the only significant independent factor that was negatively correlated with HRQOL.
• Age, sex, education, smoking history, duration of THR, and indication of THR were not significantly correlated with HRQOL.

## 1 Introduction

Total hip replacement (THR) is a commonly performed surgical procedure for advanced hip arthritis and avascular necrosis [1,2]. After THR, the ability to perform activities of daily living (ADL) generally improves, but some activities may still be challenging and difficult [[3], [4], [5], [6]]. The HRQOL have been used to objectively assess functional outcomes and document improvement after THR [7,8].

Short- and medium-term THR studies have reported substantial benefits in the generic HRQOL [9,10], About 20% of THR are done in people younger than 60 years with different diagnoses; the general increment in survival is expected to further increase the need for this procedure. These data indicate that greater attention should be paid to the long-term follow-up results of hip replacement surgery [11].

There is a scarce of data on assessment of HRQOL in patients after THR. Even less is known about possible predictors of long term outcomes of these procedures. In addition, up to the best of our knowledge there is no previous study that assessed HRQOL after THR among Iraqi patients. The goals of the current study were 1) to evaluate HRQOL after TAR in a sample of Iraqi patients, and 2) to identify possible association between HRQOL with sociodemographic and clinical characteristics of the patients.

## 2 Patients and methods

### 2.1 Study design

This observational descriptive cross sectional multicenter study was conducted at Baghdad Teaching Hospital Orthopedic Department, Specialized Surgical Hospital, AL-Dowali Private Hospital, and Private Nursing Room Hospital from April 2018 to March 2019. We measured HRQOL for patients with THR.

### 2.2 Participants

A total of 96 consecutive convenient sample of patients were enrolled in the study. Patients were eligible for the study if they had THR and their age above 18 years. Patients were excluded if they had another disease that could affect HRQOL like inflammatory arthritis, heart failure, uncontrolled respiratory diseases like chronic obstructive or interstitial or suppurative lung diseases, chronic infections like tuberculosis, or malignancy like solid tumors or hematological malignancies.

### 2.3 Data collection and evaluation

Data were collected using clinical research questionnaire form through face to face interview. Sociodemographic and clinical characteristics of the patients included age, sex, BMI, educational level, smoking status, duration and indications of THR were recorded.

### 2.4 Outcome measurements

Health related quality of life was measured using outcome measures that evaluate hip function and symptoms and included: Harris Hip score (HHS): This score has a maximum of 100 points (best possible outcome) covering pain (1 item, 0–44 points), function (7 items, 0–47 points), absence of deformity (1 item, 4 points), and range of motion (2 items, 5 points). The higher the HHS, the less dysfunction. A total score of <70 is considered a poor result; 70–80 is considered fair, 80–90 is good, and 90–100 is an excellent result [12].

### 2.5 Ethical approval and patients consent

All patients signed an informed consent form according to the principles of the Declaration of Helsinki. The local scientific ethics committee of Department of Surgery, College of Medicine, University of Baghdad approved the study protocol (no. 113). The purpose of the study was explained to each participant prior to interview and all the patients accepted to participate in the study.

### 2.6 Statistical analysis

Statistical software (SPSS version 23, IBM, USA) was used for analysis. Descriptive statistics were presented as mean ± SD for normally distributed continuous variables and frequency and percentages for categorical variables. Multiple linear regression analysis was used to assess the correlation between sociodemographic and clinical characteristics with HRQOL measured by HHS.

## 3 Results

**Figure 1:**
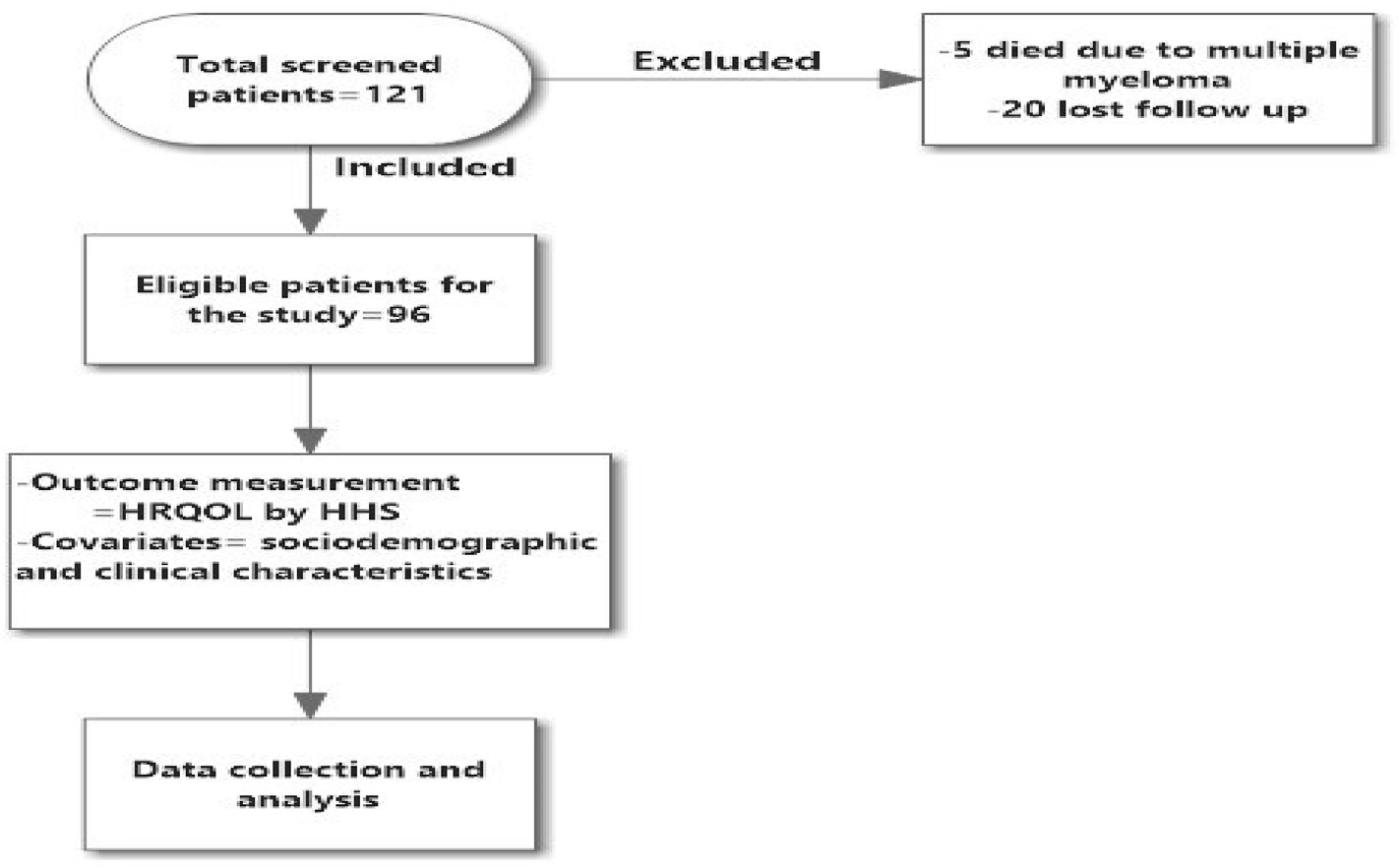
Study flow chart.

A total of 121 patients were screened for eligibility in the study. Of those 5 patients excluded due to death by multiple myeloma and 20 were lost from follow up. The eligible patients for the study were 96 patients with THR (Fig. 1). The mean age of patients was 56.76, (13.88) years with a range of 23–90 years. Most of patients were females (52 patients (54.2%). Other sociodemographic features were shown in Table 1.

**Table 1:** Sociodemographic features of patients with post hip replacement.

Variables	Value
Age Mean (SD), range, years	56.76, (13.88), 23-90
Females n(%)	52(54.2%)
BMI (SD), range, KG/m^2^	44.87(8.07), 28.100–56.700
Education level n(%)	
Illiterate	29 (30.2)
Primary	21 (21.9)
Secondary	23 (24)
College	23 (24)
Smokers n(%)	43 (44.8)
Indications n(%)	
Avascular necrosis	37(38.5)
Femoral neck fracture	28 (29.2)
Osteoarthritis	29 (30.2)
Intertrochanteric fracture	2 (2.1)
THR in years (SD) range	4.430 (2.74), 0.420–14.000

BMI, body mass index; SD, standard deviation, THR, total hip replacement.

**Fig. 2:**
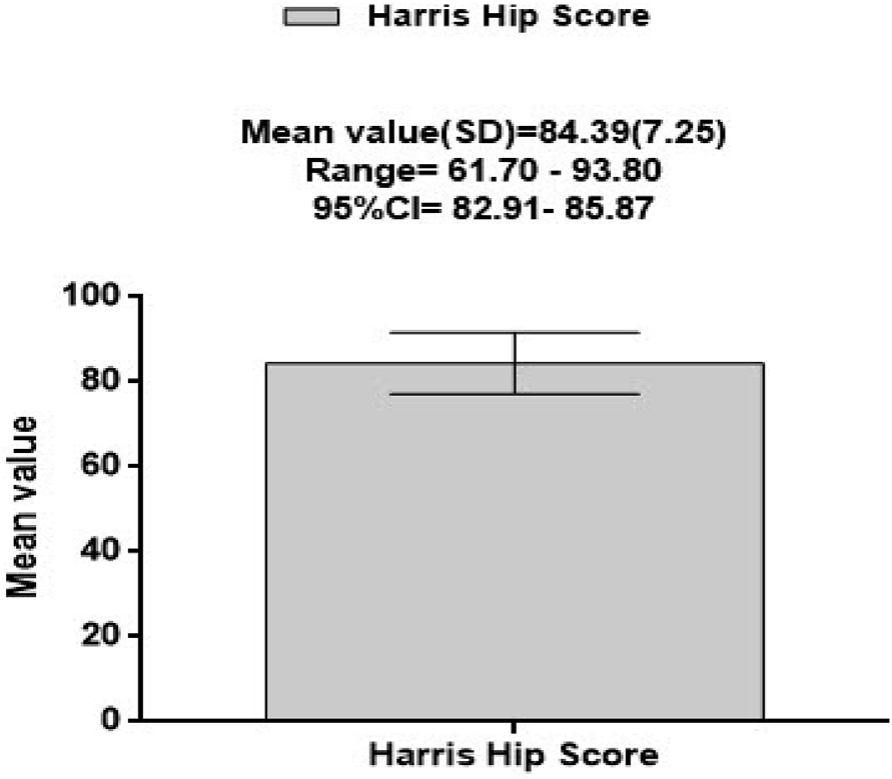
Mean quality of life (Harris Hip score) in patients post hip replacement.

The mean(SD) of HHS was: 84.389(7.2466) with minimum score of 61.7 and maximum score 93.8 and 95% CI of 82.91–85.87 (Fig. 2).

Table 2 shows the impact of sociodemographic and clinical characteristics on HRQOL measured by HHS. The only significant predictor was BMI. For each 1unit increase in BMI, there was a decrease in HHS by −0.276.

**Table 2:** Impact of sociodemographic characteristics on QOL measured by Hariss hip score.

Variables	Unstandardized regressionCoefficients		Standardized regression Coefficients	P value
	B	Std. Error	Beta	
Age	-.032	.063	-.063	.609
Sex	.208	1.708	.015	.903
BMI	-.243	.096	-.276	.013
Education	.353	.656	.059	.592
Smoking	−2.670	1.542	-.188	.087
Duration of THR, months	-.213	.270	-.082	.433
Indication	.107	1.043	.013	.919

BMI, body mass index; THR, total hip replacement.

## 4 Discussion

Up to the best our knowledge, this is the first multicenter study in Iraq that evaluated HRQOL in patients after THR and assessed the impact of sociodemographic and clinical characteristics on it and revealed that THR improve HRQOL and BMI was a significant negatively correlated independent factor with HRQOL. These findings are clinically important and indicate that patients with functional limitations and disability may get an observable benefit on hip replacement.

On reviewing literatures, similar findings were reported other studies. Rashed et al. [13] assessed the functional and clinical results after one year of cemented THR with dual mobility cup for the treatment of fracture neck femur in 31 active middle-aged patients in Egypt (Middle Eastern population) and found that mean HHS improved over the follow up period and Dual mobility cup total hip replacement was an acceptable method for treatment of displaced femoral neck fracture in active middle aged patients in Egypt as it provides pain relief and good function without compromising the stability. However our study included more number of patients (96) and different indications for hip replacement (Avascularr necrosis, advanced arthritis, femure neck fracture) and this may make our conclusions more valid.

Griffin et al. [14] published an important large study in 2015, on the recovery from hip fractures in the United Kingdom after hip replacement and reported that a steady increase in the hip function over a year of follow up as measured by the Oxford hip score.

Mariconda et al. [15] investigated Quality of life and functionality after total hip arthroplasty among 250 patients in a long-term follow-up study and concluded that patients who had undergone total hip arthroplasty have impaired long-term self-reported physical quality of life and hip functionality but they still perform physically better than untreated patients with advancedhip osteoarthritis. However, the level of post-surgical satisfaction is high.

Singh et al. [16] evaluated patient-level clinically meaningful improvements in pain and limitation of key activities of daily living (ADLs) after primary or revision THR and found that patient-level clinically meaningful improvements in pain and seven key ADLs can help patients set realistic goals for improvement after THR. Similarly Zubair et al. [17] demonstrated that THR was associated with significant improvement in quality of life.

However a recent study was in contrast to our study and found that THR was associated with remarkable reduced HRQoL in Iranian population when compared with the reference population. That explanation may be related to different sample size, study design, and the type of measurements used to assess HRQoL was SF36 [18].

The current study revealed that BMI has a negative impact on HRQOL in patients with THR. Siilar finding was reported by Laxy et al. [19] and itani et al. [20] who showed an inverse relation between BMI and HRQOL. This result should be considered to encourage patients with obesity to initiate and continue on weight-loss programs at the earliest opportunity and to design a future weight loss and weight management programs that will help in improvement of HRQL.

This study has some limitations: first: it is across sectional study and patients were assessed at one point and no follow up was done. Second, small sample size of the patients. Third, open label study. However, in spite of these limitations, this study has another strength points: It is the first multicenter study with strong inclusion and exclusion criteria that assessed HRQOL in patients with THR among Iraqi patients.

In conclusions, THR improves HRQOL and BMI was the only significant independent factor that was negatively correlated with HRQOL. This may suggest to encourage people with advanced hip arthritis, severe osteonecrosis of the hip to perform hip procedures with replacement to improve their functional state. Also to motivate obese patients to do weight reduction.

## Ethical approval

The local scientific ethics committee of Department of Surgery, College of Medicine, University of Baghdad approved the study protocol (UIN 113).

## Sources of funding

No sources of funding.

## Author contribution

Both authors(Mahmoud Khudair Yaseen and Faiq I. Gorial) contributed in concept or design of the study, data collection, data analysis or interpretation, writing the paper, and approval of the final version of the paper.

## Trial registry number

The local scientific ethics committee of Department of Surgery, College of Medicine, University of Baghdad approved the study protocol with number 113 on 2/6/2019.

Research registry UIN 4948.

Link

https://www.researchregistry.com/browse-the-registry.

## Guarantor

Faiq I. Gorial.

## Consent

All patients signed written informed consent for participation in the study.

## Declaration of competing interest

No Conflicts of interests.
